# Caveolin‐1 enhances rapid mucosal restitution by activating TRPC1‐mediated Ca^2+^ signaling

**DOI:** 10.14814/phy2.12193

**Published:** 2014-11-04

**Authors:** Navneeta Rathor, Hee K. Chung, Shelley R. Wang, Jian‐Ying Wang, Douglas J. Turner, Jaladanki N. Rao

**Affiliations:** 1 Department of Surgery, Cell Biology Group, University of Maryland School of Medicine, Baltimore, Maryland, USA; 2 Baltimore VA Medical Center, Baltimore, Maryland, USA; 3 Department of Pathology, University of Maryland School of Medicine, Baltimore, Maryland, USA

**Keywords:** Ca^2+^ influx, Cav^−/−^mice, cell migration, cyclopiazonic acid, hypertonic NaCl injury, intracellular Ca^2+^, TRPC1

## Abstract

Early rapid mucosal restitution occurs as a consequence of epithelial cell migration to reseal superficial wounds, a process independent of cell proliferation. Our previous studies revealed that the canonical transient receptor potential‐1 (TRPC1) functions as a store‐operated Ca^2+^ channel (SOCs) in intestinal epithelial cells (IECs) and regulates epithelial restitution after wounding, but the exact mechanism underlying TRPC1 activation remains elusive. Caveolin‐1 (Cav1) is a major component protein that is associated with caveolar lipid rafts in the plasma membrane and was recently identified as a regulator of store‐operated Ca^2+^ entry (SOCE). Here, we showed that Cav1 plays an important role in the regulation of mucosal restitution by activating TRPC1‐mediated Ca^2+^ signaling. Target deletion of Cav1 delayed gastric mucosal repair after exposure to hypertonic NaCl in mice, although it did not affect total levels of TRPC1 protein. In cultured IECs*,* Cav1 directly interacted with TRPC1 and formed Cav1/TRPC1 complex as measured by immunoprecipitation assays. Cav1 silencing in stable TRPC1‐transfected cells by transfection with siCav1 reduced SOCE without effect on the level of resting [Ca^2+^]_cyt_. Inhibition of Cav1 expression by siCav1 and subsequent decrease in Ca^2+^ influx repressed epithelial restitution, as indicated by a decrease in cell migration over the wounded area, whereas stable ectopic overexpression of Cav1 increased Cav1/TRPC1 complex, induced SOCE, and enhanced cell migration after wounding. These results indicate that Cav1 physically interacts with and activates TRPC1, thus stimulating TRPC1‐mediated Ca^2+^ signaling and rapid mucosal restitution after injury.

## Abstract (precis)

Early rapid mucosal restitution occurs as a consequence of epithelial cell migration to reseal superficial wounds, a process independent of cell proliferation. Caveolin‐1 (Cav1) is a major component protein that is associated with caveolar lipid rafts in the plasma membrane and was recently identified as a regulator of store‐operated Ca^2+^ entry (SOCE). Our results indicate that Cav1 physically interacts with and activates TRPC1, thus stimulating TRPC1‐mediated Ca^2+^ signaling and rapid mucosal restitution after injury.

## Introduction

Epithelial cells line the gastrointestinal mucosa and form an important barrier to a wide variety of noxious substances and invasive enteric pathogens in the lumen. Early mucosal restitution refers to the resealing of superficial wounds to this barrier as a consequence of epithelial cell migration into the defect, a process independent from epithelial cell proliferation (Rutten and Ito 1983; Wang and Johnson 1991; Nusrat et al. 1992; Dignass et al. 1994). This rapid mucosal reepithelialization after superficial wounding is a complex process that is tightly regulated by numerous factors, but its exact mechanism remains still unclear. A significant body of evidence indicates that cytosolic free Ca^2+^ ([Ca^2+^]_cyt_) plays an important role in the regulation of intestinal epithelial cells (IEC) migration after injury and that increasing [Ca^2+^]_cyt_ stimulates epithelial restitution (Rao et al. 2001, 2002, 2003, 2006, 2007, 2008; Rao and Wang 2002, 2006; Rathor et al. 2014). Ca^2+^ entry due to store depletion is often called capacitative or store‐operated Ca^2+^ entry (SOCE) and is mediated by Ca^2+^‐permeable channels termed store‐operated Ca^2+^ channels (SOCs), which contributes to the sustained increase in [Ca^2+^]_cyt_ and the refilling of Ca^2+^ into the stores. Voltage‐gated K^+^ (Kv) channels regulate Ca^2+^ influx by regulating the membrane potential (*E*_m_) that governs the driving force for Ca^2+^ influx in IECs (Wang et al. 2000; Rao et al. 2002; Rao and Wang 2006). Our recent studies show that canonical transient receptor potential‐1 (TRPC1) is a candidate protein for Ca^2+^‐permeable channels mediating store‐operated Ca^2+^ entry (SOCE) in IECs and plays an important role in early epithelial restitution after injury (Rao et al. 2006, 2010; Rao and Wang 2011). However, the exact signals initiating TRPC1 activation after mucosal injury remain unknown.

Recently, several studies have demonstrated that key molecules involved in Ca^2+^ signaling are associated with caveolar lipid rafts, thus implicating the importance of caveolae in Ca^2+^ signaling (Lockwich et al. 2000; Prakash et al. 2007). Caveolae are flask‐shaped plasma membrane (PM) invaginations in different cell types and are known to express a ~22 kDa protein, Caveolin‐1 (Cav1) (Isshiki et al. 2002; Liu et al. 2002). Cav1 is a multifunctional scaffolding protein and its various binding partners associate with many aspects of cellular processes ranging from cholesterol homeostasis, vesicular transport, cell cycle and cell polarity, to cell transformation and signal transduction (Isshiki et al. 2002; Liu et al. 2002; Grande‐García et al. 2007). It has been shown that Ca^2+^ influx occurs via caveolae in response to ER‐stored Ca^2+^ depletion in different types of cells (Kwiatek et al. 2006; Adebiyi et al. 2011). The Caveolin scaffolding domain (CSD) of Cav1 allows for the physical and functional interaction with Ca^2+^‐permeable channels within the caveolae (Lockwich et al. 2000; Sundivakkam et al. 2009; Adebiyi et al. 2011). The presence of TRPC1 in these caveolar lipid raft domains constitutes an important factor in its function as a SOC. In fact, the binding of Cav1 to both the NH_2_ and COOH termini of TRPC1 is necessary for caveolar distribution of TRPC1 (Lockwich et al. 2000; Sundivakkam et al. 2009), and increased Cav1 expression is associated with enhanced Ca^2+^ entry in response to Ca^2+^ store depletion (Patel et al. 2007). Disruption of caveolar proteins, either with chemical inhibitors or silencing the Cav1 gene, is shown to inhibit SOCE (Isshiki et al. 1998; Mercier et al. 2009), whereas restoration of wild‐type (WT) Cav1 in Cav1 knockout (Cav1^−/−^) cells rescued SOCE (Grande‐García et al. 2007).

In this study, we tested the hypothesis that Cav1 regulates rapid gut mucosal restitution by modulating TRPC1‐mediated Ca^2+^ signaling after injury in vivo and in vitro models. First, we investigated whether target deletion of the *Cav1* gene delayed gastric mucosal repair in mice after exposure to hypertonic NaCl. Second, we examined the expression pattern of Cav1 and its physical interaction with TRPC1 in gastric mucosal tissue and cultured IECs. Third, we investigated whether Cav1 silencing decreased Cav1/TRPC1 interactions, altered SOCE, and inhibited cell migration in stable TRPC1‐transfected cells (IEC‐TRPC1). Finally, we determined whether ectopic overexpression of Cav1 increased Cav1/TRPC1 associations and promoted cell migration after wounding.

## Materials and Methods

### Chemicals and supplies

Disposable culture ware was purchased from Corning Glass Works (Corning, NY). Tissue culture media, insulin, gentamicin, isopropyl‐*β*‐d‐thiogalactopyranoside (IPTG), LipofectAMINE 2000, and fetal bovine serum (FBS) were obtained from Invitrogen (Carlsbad, CA), and other biochemicals were obtained from Sigma (St. Louis, MO). The affinity‐purified rabbit polyclonal antibody against TRPC1 was purchased from Alomone Laboratories (Jerusalem, Israel), and antibody against Cav1 was from Cell Signaling Technology (Danvers, MA). Actin antibody that recognizes all isoforms (*α*,* β* and *γ*) was purchased from EMD Millipore (Bedford, MA) (Cat#CP‐01).

### Animals and procedures

All animal studies were conducted according to a protocol approved by the Institutional Animal Care and Use Committee of University of Maryland School of Medicine and Baltimore VA Medical Center. All procedures were confirmed to NIH animal welfare guidelines. Cav1‐deficient mice strain (Cav1^tm1Mls^/J; Cav1^−/−^) and their control (wild‐type) littermates (Cav1^+/+^) were obtained from The Jackson Laboratory (Bar Harbor, ME). Mice were housed and maintained in a barrier facility at our Baltimore VA Medical Center. Pathogen‐free procedures are used in all mouse rooms. Mice were kept on a 12:12‐h light–dark cycle with ad libitum access to food and water until experiments were conducted. Animals were deprived of food but allowed free access to water for 22 h before the experiments. Both Cav1^−/−^ and control littermate mice were divided into different groups and each study was performed using four to five mice per group. Mice were administered with 0.2 mL of 3.4 mol/L NaCl intragastrically, as described previously (Coimbra et al. 1997; Tatemichi et al. 2003). The animals were euthanized by anesthesia with CO_2_ asphyxiation at 3, 6, and 16 h after hypertonic NaCl administration. Control mice received 0.2 mL of isotonic saline intragastrically. The stomachs were removed, opened along the greater curvature, and rinsed in ice‐cold saline. They were laid flat on a Petri dish inverted over ice, and examined for gross damage (macroscopic). The method described by Takagi and Okabe (1968) was used to determine the severity of lesions. The incidence of lesions was noted, and the length of the visible lesions was measured. The macroscopic ulcer index was expressed as total lesion length in millimeters. Immediately following measurement, surface area of the stomach oxyntic gland mucosa was scraped away from the underlying smooth muscle with a glass slide. Mucosal scrapings were employed to extract total proteins using standardized procedures (Wang and Johnson 1991; Rao et al. 2001, 2002, 2008) for immunoprecipitation (IP) and immunoblotting analysis.

### Cell culture

The line of IEC‐6 cells was purchased from the American Type Culture Collection (ATCC) at passage 13. IEC‐6 cells were derived from normal rat intestinal crypt cells and were developed and characterized by Quaroni et al. (Quaroni et al. 1979). Stock cells were maintained in T‐150 flasks in Dulbecco's modified Eagle medium (DMEM) supplemented with 5% heat‐inactivated FBS, 10 *μ*g/mL insulin, and 50 *μ*g/mL gentamicin sulfate. Flasks were incubated at 37°C in a humidified atmosphere of 90% air‐10% CO_2_, and passages 15–20 were used in the experiments. Stable *Cdx2*‐transfected IEC‐6 cells were developed and characterized by Suh and Traber (Suh and Traber 1996) and were a kind gift from Dr. Peter G. Traber (Baylor College of Medicine, Houston, TX). Stock‐stable *Cdx2*‐transfected IEC‐6 (IEC‐Cdx2L1) cells were grown in DMEM supplemented with 5% heat‐inactivated FBS, 10 *μ*g/mL insulin, and 50 *μ*g/mL gentamicin sulfate. Before experiments, IEC‐Cdx2L1 cells were grown in DMEM containing 4 mmol/L IPTG for 16 days to induce cell differentiation as described in our earlier publications (Rao et al. 1999, 2000, 2002, 2007; Rathor et al. 2014). Since the rapid mucosal restitution of superficial wounds in vivo is the function of differentiated IECs from the surface of the mucosa rather than from undifferentiated IECs from within crypts, differentiated IEC‐Cdx2L1 cells were chosen as a model in this study. The stable TRPC1‐transfected IEC‐6 cells (IEC‐TRPC1) were developed and characterized as described in our earlier publications (Rao et al. 2006, 2010) and cultured in DMEM medium used for growing IEC‐6 cells.

### RNA interference

The small interfering (si)RNA that were designed to specifically target the coding region of Cav1 (siCav1) mRNA was purchased from Dharmacon Inc (Lafayette, CO). Scrambled control siRNA (C‐siRNA), which had no sequence homology to any known genes, was used as the control. The siCav1 and C‐siRNA were transfected into cells as described previously (Rao et al. 2006, 2010; Zhuang et al. 2013). Briefly, for each 60‐mm cell culture dish, 20 *μ*L of the 5 *μ*mol/L stock siCav1, or C‐siRNA was mixed with 500 *μ*L of Opti‐MEM medium (Invitrogen). This mixture was added to a solution containing LipofectAMINE 2000 in 500 *μ*L of Opti‐MEM. The solution was incubated for 20 min at room temperature and gently overlaid onto monolayers of cells in 3 mL of medium, and cells were harvested for various assays after 48‐h incubation.

### Plasmid construction and transfection

The transfection grade eukaryotic expression vector pCMV6‐Neo containing the full‐length cDNA of human *Cav1* (~2.4 kb) gene under the control of cytomegalovirus (CMV) promoter and its control vector lacking Cav1 cDNA (Null) were purchased from Origene Technologies (Rockville, MD). The Cav1 cDNA was inserted into the Not1 multiple cloning sites of the pCMV6‐Neo vector. In order to obtain large quantities of cDNA for our experiments, we have transformed into DH5*α* bacteria, and resulting clones were sequenced for the confirmation Cav1 cDNA insertion. The IEC‐6 cells were transfected with the Cav1 expression vector or control vector using the LipofectAMINE 2000 and performed as recommended by the manufacturer (Invitrogen). After the 5‐h period of incubation, the transfection medium was replaced by the standard growth medium containing 5% FBS for 2 days before exposure to the selection medium. These transfected cells were selected for Cav1 integration by incubation with the selection medium containing 0.6 mg/mL of G418, and clones resistant to the selection medium were isolated, cultured, and screened for Cav1 expression by western blot analysis with the specific anti‐Cav1 antibody.

### Immunoprecipitation (IP) and immunoblotting analysis

Cell samples, dissolved in ice‐cold RIPA‐buffer (50 mmol/L Tris/HCl, pH 7.4, 150 mmol/L NaCl, 1 mmol/L DTT, 0.5 mmol/L EDTA, 1.0% NP40, 0.5% sodium deoxycholate, 0.1% SDS, 2 mmol/L phenylmethyl‐sulfonyl fluoride, 20 *μ*g/mL aprotinin, 2 *μ*g/mL leupeptin, and 2 mmol/L sodium orthovanadate), were sonicated and centrifuged at 4°C, and the supernatants were collected for IP. Equal amounts of proteins (500 *μ*g) for each sample were incubated with the specific antibody against Cav1 or TRPC1 (4 *μ*g) at 4°C for 3 h, and protein A/G‐PLUS‐Agarose was added and incubated overnight at 4°C. The precipitates were washed five times with ice‐cold D‐PBS, and the beads were resuspended in SDS sample buffer. For immunoblotting, samples were subjected to electrophoresis on PAGE gels described previously (Rao et al. 2008; Xiao et al. 2013; Zhuang et al. 2013). Briefly, after the transfer of protein onto nitrocellulose membranes, the membranes were incubated for 1 h in 5% nonfat dry milk in 1× TBS‐T buffer (Tris‐buffered saline, pH 7.4, with 0.1% Tween‐20). Immunologic evaluation was then performed overnight at 4°C in 5% nonfat dry milk/TBS‐T buffer containing a specific antibody against Cav1 or TRPC1. The membranes were subsequently washed with 1× TBS‐T and incubated with the secondary antibodies conjugated with horseradish peroxidase for 1 h at room temperature. The immunocomplexes on the membranes were reacted for 1 min with Chemiluminescence Reagent (NEL‐100 DuPont NEN).

### Measurement of [Ca^2+^]_cyt_

Detailed digital imaging methods employed for measuring [Ca^2+^]_cyt_ were described in our previous publications (Rao et al. 2006, 2007, 2008, 2010, 2012; Rathor et al. 2014). Briefly, cells were plated on 25‐mm coverslips and incubated in culture medium containing 3.3 *μ*mol/L fura‐2 AM for 30 min at room temperature (22–24°C) under an atmosphere of 10% CO_2_ in air. The fura‐2 AM‐loaded cells were then superfused with standard bath solution for 20–30 min at 22–24°C to wash away extracellular dye and permit intracellular esterases to cleave cytosolic fura‐2 AM into active fura‐2. Fura‐2 fluorescence from the cells and background fluorescence were imaged using a Nikon Diaphot microscope equipped for epifluorescence. Fluorescent images were obtained using a microchannel plate image intensifier (Amperex XX1381; Opelco, Washington, DC) coupled by fiber optics to a Pulnix charge‐coupled device video camera (Stanford Photonics, Stanford, CA). Image acquisition and analyses were performed with a Metamorph Imaging System (Universal Imaging). The final values of [Ca^2+^]_cyt_ were obtained from fura‐2 fluorescent emission excited at 380 and 340 nm from calibrated ranges as described in our previous publications (Rao et al. 2007, 2008; Rao and Wang 2011; Rathor et al. 2014).

### Measurement of cell migration

Migration assays were carried out as described in our earlier publications (Rao et al. 2001, 2002, 2003, 2006, 2007, 2008; Rao and Wang 2002, 2006; Zhuang et al. 2013; Rathor et al. 2014). Cells were plated at 6.25 × 10^4^/cm^2^ in DMEM containing FBS on 60‐mm dishes thinly coated with Matrigel according to the manufacturer's instructions (BD Biosciences, Bedford, MA) and were incubated as described for stock cultures. Cells were fed on day 2, and cell migration was assayed on day 4. To initiate migration, the cell layer was scratched with a single edge razor blade cut to ~27 mm in length. The scratch was made over the diameter of the dish and extended over an area 7–10 mm wide. The migrating cells in six contiguous 0.1‐mm squares were counted at ×100 magnification beginning at the scratch line and extending as far out as the cells had migrated. All experiments were carried out in triplicates, and the results were reported as the number of migrating cells per millimeter of scratch.

### Statistical analysis

All data are expressed as means ± SE from four to five mice per group. IP and immunoblotting results were repeated three times. The significance of the difference between means was determined by analysis of variance (ANOVA). The level of significance was determined using the Duncan's multiple‐range test (Harter 1960) and values of *P* < 0.05 were considered significant.

## Results

### Target deletion of the Cav1 gene delays gastric mucosal repair after injury

**Figure 1.**
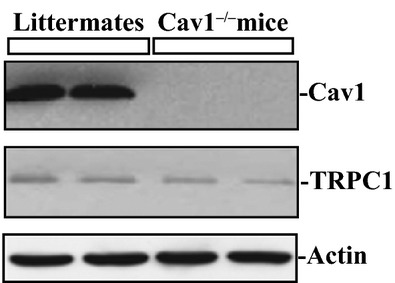
Cav1 and TRPC1 protein expression in gastric mucosa isolated from Cav1^−/−^ mice and control wild‐type littermates. Levels of Cav1 and TRPC1 were examined by western blot analysis. Actin immunoblotting was performed as an internal control for equal loading. Three separate experiments were performed that showed similar results.

To determine the in vivo function of Cav1 in the regulation of gut mucosal restitution, Cav1^−/−^ mice were used in this study. First, we characterized Cav1^−/−^ mice to verify the basal levels of Cav1 and TRPC1 proteins in the gastric mucosa. Heterozygous Cav1^+/−^ mice appeared phenotypically normal and were subsequently intercrossed for the generation of homozygous Cav1^−/−^ mice. Generally, Cav1^−/−^ mice looked normal; there were no significant differences in body weight, gastrointestinal gross morphology, and general appearances between Cav1^−/−^ mice and littermate controls (data not shown). Age‐matched Cav1^−/−^ mice and littermate control mice (3 months old) were used for phenotype analysis. Mucosal scrapings were isolated and analyzed by western immunoblotting assays using specific anti‐Cav1 or TRPC1 antibody. As shown in Figure 1, gastric mucosa expressed a very high basal level of Cav1 protein in control wild‐type littermates, but Cav1 protein was undetectable in the mucosa in Cav1^−/−^ mice, as expected. However, there were no significant changes in basal levels of TRPC1 expression in the gastric mucosa between control littermate and Cav1^−/−^ mice.

**Figure 2.**
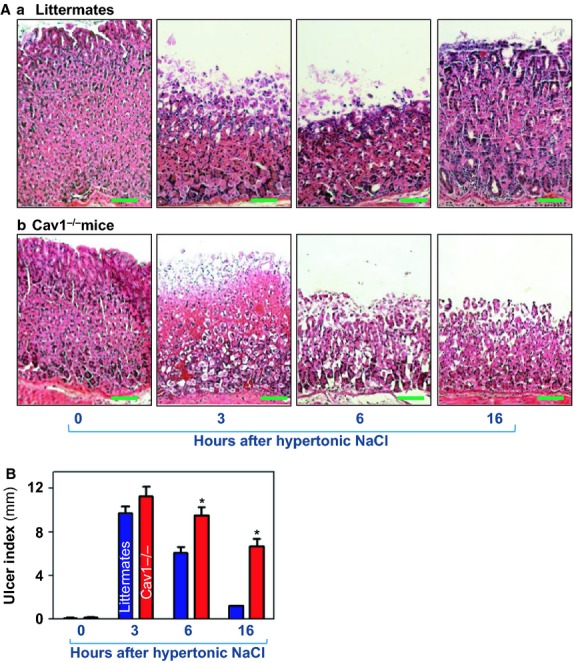
Histological appearance and macroscopic damage of gastric mucosa after exposure to hypertonic NaCl in mice. Gastric mucosal injury was induced by intragastric administration of 3.4 mol/L NaCl as described in Material and Methods section. Mice were euthanized during recovery from injury at 0, 3, 6, and 16 h after the administration of NaCl. (A) Paraffin‐embedded sections with H/E staining from different times after injury. Scale bar, 100 *μ*m. Original magnification ×100. (B) Ulcer index in gastric mucosa after injury. Values are means ± SEM from four to five mice/group. *P < 0.05 compared with control littermates.

Second, we examined changes in the rate of gastric mucosal repair and the levels of Cav1/TRPC1 complexes in the mucosal tissue after exposure to hypertonic NaCl as described previously (Coimbra et al. 1997; Tatemichi et al. 2003). Exposure of the gastric mucosa to 3.4 mol/L NaCl induced visible lesions in littermate and Cav1^−/−^ mice. Histological examination (Fig. 2A) and macroscopic damage (Fig. 2B) showed that the maximal mucosal injury occurred 3 h after intragastric administration of hypertonic NaCl and that the repair process began 6 h and was almost completed 16 h in littermate mice. However, target deletion of the *Cav1* gene significantly delayed gastric mucosal repair of hypertonic NaCl‐induced injury. We have also examined changes in mucosal injury at 1 and 2 h after the exposure to NaCl and found no significant damage to gastric mucosa (data not shown). At 16 h after administration of hypertonic NaCl, visible lesions in the gastric mucosa were still noticeable in Cav1^−/−^ mice (Fig. 2Ab, right panel), indicating the importance of Cav1 in normal mucosal repair after injury.

**Figure 3.**
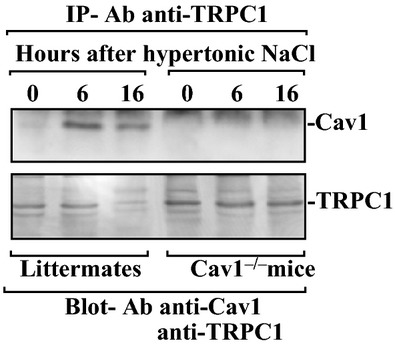
Levels of Cav1, TRPC1, and their complexes in gastric mucosa after hypertonic NaCl‐induced injury in mice. After cell lysates (500 *μ*g) were immunoprecipitated (IP) by the specific Ab against TRPC1, precipitates were subjected to SDS‐PAGE (10% acrylamide). Levels of Cav1 and TRPC1 proteins were measured using western blot analysis with the antibody against Cav1 or TRPC1. Three separate experiments were performed that showed similar results.

Third, we examined the association of Cav1 with TRPC1 in the extracts of gastric mucosa during mucosal repair after hypertonic NaCl‐induced injury. As shown in Figure 3, Cav1/TRPC1 complexes as measured by IP with anti‐TRPC1 antibody increased significantly 6 h after the administration of hypertonic NaCl and this increase was continued until 16 h in control littermate mice. In contrast, Cav1/TRPC1 complex in the gastric mucosa was undetectable in Cav1^−/−^ mice during repair after injury. These results clearly indicate that Cav1 plays a critical role in mucosal repair after injury through a process involving TRPC1.

### Interaction of Cav1 with TRPC1 in cultured IECs

**Figure 4.**
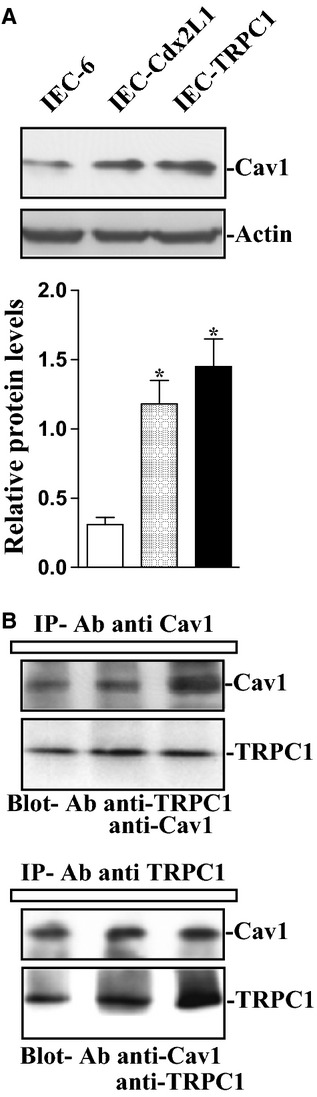
Levels of Cav1 and its interaction with TRPC1 in different lines of IECs. (A) representative immunoblot of Cav1 in IEC‐6 cells, differentiated IEC‐Cdx2L1 cells, and IECs stably overexpressing TRPC1 (IEC‐TRPC1). Levels of total Cav1 were examined by western blot analysis, and actin immunoblotting was performed as an internal control for equal loading (upper panel). Quantitative analysis of western immunoblots by densitometry that were corrected for actin loading from cells described above. Values are means ± SEM;* P* < 0.05 compared with parental IEC‐6 cells (lower panel). (B) levels of Cav1 and TRPC1 in the complex IP by the anti‐Cav1 or anti‐TRPC1 Ab in cells described in (A). Three separate experiments were performed that showed similar results.

Our previous studies have shown that TRPC1 functions as SOC in IECs and mediates Ca^2+^ influx after store depletion (Rao et al. 2006, 2010). To further determine the possibility that Cav1 regulates Ca^2+^ influx through its interaction with TRPC1, basal levels of Cav1 and its interaction with TRPC1 were examined in three lines of IECs including IEC‐6, differentiated IEC‐Cdx2L1, and stable TRPC1‐transfected cells (IEC‐TRPC1). As shown in Figure 4A, differentiated IEC‐Cdx2L1 and IEC‐TRPC1 cells expressed higher levels of Cav1 compared to that observed in IEC‐6 cells. Both IEC‐Cdx2L1 and IEC‐TRPC1 cells exhibited four‐fold increase of IEC‐6 cell in the level of Cav1 protein, which was associated with increased Ca^2+^ influx after store depletion as reported previously (Rao et al. 2006, 2010). To determine whether Cav1 forms Cav1/TRPC1 complexes in different IECs, whole‐cell lysates were immunoprecipitated (IP) with either anti‐Cav1 or anti‐TRPC1 antibody, and then these precipitates were examined by western blot analysis using specific antibody against TRPC1 or Cav1. As shown in Figure 4B, IP of Cav1 or TRPC1 resulted in co‐IP of TRPC1 and Cav1 in all three lines of IECs, although the levels of TRPC1 protein in IEC‐TRPC1 cells were higher than those observed in IEC‐6 and IEC‐Cdx2L1 cells. We also used IgG as a negative control in IP assays and found that incubation with IgG at the same condition did not pull down either Cav1 or TRPC1 (data not shown). These results indicate that Cav1 physically interacts with TRPC1 in various IECs.

### Cav1 silencing decreases SOCE and represses epithelial restitution in stable IEC‐TRPC1 cells

**Figure 5.**
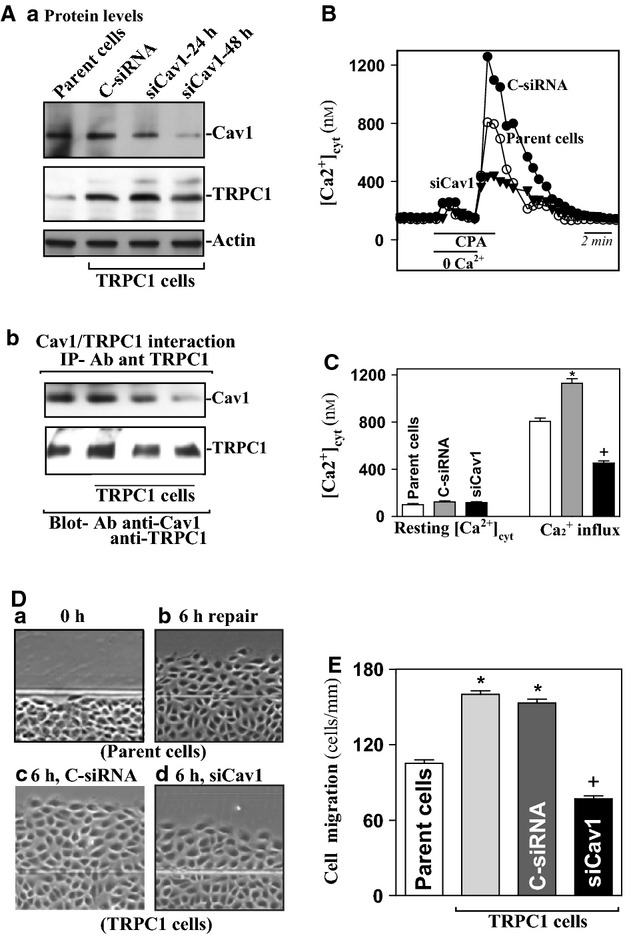
Effect of Cav1 silencing on Cav1/TRPC1 complex, SOCE, and cell migration in stable IEC‐TRPC1 cells. (Aa) representative Cav1 and TRPC1 immunoblots. After cells were transfected with either siRNA targeting the Cav1 mRNA coding region (siCav1) or control siRNA (C‐siRNA) for 24 and 48 h, whole‐cell lysates were harvested for western blot analysis to monitor the expression of Cav1, TRPC1 and loading control actin. (Ab) changes in the levels of Cav1/TRPC1 protein in the complex IP by anti‐TRPC1 antibody in cells described in (Aa). (B) representative records showing the time course of [Ca^2+^]_cyt_ changes after exposure to 10 *μ*mol/L cyclopiazonic acid (CPA) in the absence (0Ca^2+^) or presence of extracellular Ca^2+^ in parent IEC‐6 cells and IEC‐TRPC1 cells transfected with C‐siRNA or siCav1 for 48 h. (C) summarized data showing resting [Ca^2+^]_cyt_ (*left*) and the amplitude of CPA‐induced Ca^2+^ influx (*right*) from cells described in (B). Values are means ± SEM;* n* = 25. *P < 0.05 compared with parent IEC‐6 cells; ^+^*P* < 0.05 compared with cells transfected with C‐siRNA. (D) images of cell migration after wounding: (a) 0 h after wounding; (b) 6 h after wounding in parent IEC‐6 cells; (c) 6 h after wounding in IEC‐TRPC1 cells transfected with C‐siRNA; and (d) 6 h after wounding in IEC‐TRPC1 cells transfected with siCav1 for 48 h. (E) summarized data showing rates of cell migration after wounding in cells described in (D). Data were expressed as means ± SEM from six dishes. *P < 0.05 compared with parent IEC‐6 cells;^+^*P* < 0.05 compared with cells transfected with C‐siRNA.

Our previous study shows that ectopic TRPC1 overexpression increases SOCE and stimulates IEC migration after wounding (Rao et al. 2006). In this study, we further determined if Cav1 is necessary for TRPC1‐mediated Ca^2+^ influx during restitution after wounding. siRNA targeting Cav1 mRNA (siCav1) was used to specifically block endogenous Cav1 in stable IEC‐TRPC1 cells. Initially, we determined the transfection efficiency of the siRNA nucleotides and demonstrated that more than 95% of IEC‐TRPC1 cells were positive when they were transfected with a fluorescent FITC‐conjugated C‐siRNA for 48 h (data not shown). As shown in Figure 5Aa, transfection with siCav1 for 48 h decreased Cav1 protein levels by ~90%, but it did not affect TRPC1 content (relative protein levels from 1.4 ± 0.17 in C‐siRNA to 0.21 ± 0.018 in 48 h siCav1; *P* < 0.05). Transfection with control siRNA (C‐siRNA) at the same concentrations showed no significant effect on Cav1 level. Cav1 silencing by siCav1 reduced Cav1/TRPC1 complexes as measured by IP assays (Fig. 5Ab). Although Cav1 silencing had no effect on the levels of resting [Ca^2+^]_cyt_, it significantly inhibited cyclopiazonic acid (CPA)‐induced Ca^2+^ influx in stable IEC‐TRPC1 cells (Fig. 5B and C). The level of Ca^2+^ influx after store depletion was decreased by ~70% in Cav1‐silenced cells compared with those observed in either parent IEC‐6 or cells transfected with C‐siRNA (from 1129 ± 96 nmol/L in C‐siRNA to 452 ± 47 nmol/L in 48 h siCav1; *n*= 25, *P* < 0.05). Cav1 silencing also impaired epithelial restitution after wounding in stable IEC‐TRPC1 cells (Fig. 5D and E). The number of cells migrating over the denuded area 6 h after wounding was decreased by ~40% in Cav1‐silenced cells. We also examined the effect of Cav1 silencing on Ca^2+^ influx and epithelial restitution in other lines of IECs and demonstrated that decreased levels of Cav1 by transfection with siCav1 also inhibited SOCE and repressed cell migration after wounding in differentiated IEC‐Cdx2L1 cells (data not shown). In addition, neither siCav1 nor C‐siRNA affected cell viability as measured by Trypan blue staining (data not shown). These findings strongly indicate that Cav1 is crucial for stimulation of cell migration after wounding by activating TRPC1‐mediated Ca^2+^ signaling.

### Ectopic overexpression of the Cav1 gene increases SOCE and promotes cell migration

**Figure 6.**
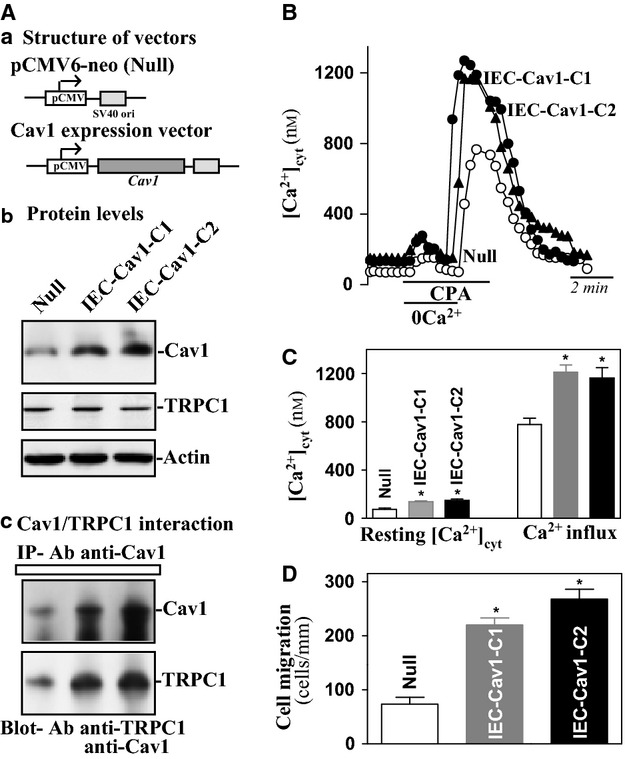
Effect of ectopic overexpression of Cav1 on the levels of Cav1, SOCE, and cell migration after wounding. (Aa) structure of expression vector. (Ab) representative Cav1 and TRPC1 immunoblots in two different clones (C1 and C2) of stable Cav1‐transfected cells (IEC‐Cav1). IEC‐6 cells were transfected with the Cav1 expression vector or control empty vector (Null), and clones resistant to the selection medium containing 0.6 mg/mL G418 were isolated and screened for Cav1 and TRPC1 expression. (Ac) changes in the levels of Cav1 and TRPC1 in the complex IPed by anti‐Cav1 Ab in cells described in (Ab). Levels of TRPC1 and Cav1 were measured using western blot analysis. (B) representative records showing the time course of [Ca^2+^]_cyt_ changes after exposure to 10 *μ*mol/L CPA in the absence (0Ca^2+^) or presence of extracellular Ca^2+^ in cells described in (Ab). (C) summarized data showing resting [Ca^2+^]_cyt_ (*left*) and the amplitude of CPA‐induced Ca^2+^ influx (*right*) from cells described in (B). Values are means ± SEM;* n* = 25. *P < 0.05 compared with cells transfected with the Null. (D) summarized data showing cell migration 6 h after wounding in cells described in (Ab). Values are means ± SEM from six dishes. *P < 0.05 compared with cells transfected with the Null.

To further define the role of Cav1 in Ca^2+^ influx in IECs, stable Cav1‐transfected IEC‐6 cells (IEC‐Cav1) were developed in this study. The expression vector encoding the full‐length cDNA of the human *Cav1* under the control of the CMV promoter was constructed as shown in Figure 6Aa. Two clones that were resistant to the selection medium containing 0.6 mg/mL G418 were characterized by examining the levels of Cav1 protein. Levels of Cav1 protein in stable IEC‐Cav1 cells were approximately five‐fold of the levels in IEC‐6 cells transfected with the control vector containing no Cav1 cDNA (Null) (relative protein levels from 0.55 ± 0.07 in Null to 2.41 ± 0.3 in IEC‐Cav1‐C1; *P* < 0.05). On the other hand, there were no differences in the levels of TRPC1 between IEC‐Cav1 and Null cells. To determine if increased levels of Cav1 enhanced Cav1/TRPC1 complex, whole‐cell lysates were immunoprecipitated with the specific anti‐Cav1 antibody. As shown in Figure 6Ac, levels of Cav1/TRPC1 complexes were higher in IEC‐Cav1 cells than those observed in Null cells. Consistently, IEC‐Cav1 cells exhibited an increase in SOCE and cell migration after wounding. Levels of resting [Ca^2+^]_cyt_ and SOCE in IEC‐Cav1 cells were increased by ~60% compared with Null cells (Fig. 6B and C), and the number of cells migrating over the wounded edge in IEC‐Cav1 cells were increased by ~55%. Increased migration in IEC‐Cav1 cells is not simply due to clonal variation, since identical results were observed when two independently transfected clones (IEC‐Cav1‐C1, IEC‐Cav1‐C2) were analyzed. These results indicate that ectopic overexpression of the *Cav1* gene activates TRPC1‐mediated Ca^2+^ signaling and promotes epithelial restitution after wounding.

## Discussion

In response to acute mucosal injury in the gut, damaged IECs are sloughed, and remaining viable cells migrate from areas adjacent to or just beneath the injured surface to cover the denuded area in vivo (Silen and Ito 1985; Wang and Johnson 1990; Hines et al. 2000), but the exact mechanism underlying this process remains largely unknown. In this study, we established the novel function of Cav1 in the control of epithelial restitution after wounding, thus advancing our understanding of the mechanism by which gut mucosa repairs itself rapidly after acute injury under physiological and various pathological conditions. Experiments aimed at characterizing the molecular aspects of Cav1 in early epithelial restitution indicates that Cav1 directly interacts with and activates TRPC1 in IECs and that formation of the Cav1/TRPC1 complex is crucial for the stimulation of Ca^2+^ influx through SOCE and cell migration after wounding.

One of the major findings reported in this study is that early mucosal repair was delayed significantly as determined by histological examination and macroscopic ulcer index (Fig. 2) when the *Cav1* gene was globally deleted in mice. Target deletion of the *Cav1* gene reduced Cav1/TRPC1 complexes, thus contributing to the delayed repair of mucosal erosions in Cav1^−/−^ mice. Our findings are consistent with studies from others who showed that wound healing in the skin in Cav1^−/−^ mice was significantly impaired compared to littermates (Grande‐García et al. 2007). Moreover, fibroblasts isolated from Cav1^−/−^ mice also exhibited a significant reduction in cell polarization and impaired cell migration (Beardsley et al. 2005; Grande‐García et al. 2007; Grande‐Garcia and del Pozo 2008). To our knowledge, these results are the first report showing that Cav1 associates with TRPC1 in the gastric mucosa and plays a critical role in the maintenance of gut epithelial integrity.

Another finding reported here is that the increased Cav1 levels in differentiated IEC‐Cdx2L1 and stable IEC‐TRPC1 cells were associated with increases in store‐depletion‐induced Ca^2+^ influx and cell migration after wounding. Our result showed that Cav1 physically interacted with TRPC1 and formed the Cav1/TRPC1 complex in IECs, whereas Cav1 silencing reduced Cav1/TRPC1 association, decreased SOCE, and repressed cell migration after wounding in stable IEC‐TRPC1 cells. Cav1 is a multifunctional scaffolding protein consisting of multiple binding sites that associate with lipid raft domains in the plasma membrane (PM) (Lockwich et al. 2000; Liu et al. 2002) and is shown to contribute to the assembly of SOCs by regulating PM localization of Ca^2+^‐permeable channels in HSG and MDCK cells (Lockwich et al. 2000; Brazer et al. 2003; Prakash et al. 2007; Adebiyi et al. 2011). The TRPC1 is predominantly localized within cholesterol‐rich lipid rafts termed caveolae microdomains, while Ca^2+^ influx occurs in response to store depletion in the endoplasmic reticulum (ER) of endothelial cells (Kwiatek et al. 2006). Studies from Sundivakkam et al. showed that the Cav1 scaffolding domain (NH_2_‐terminal residues 82–101; CSD) directly interacts with TRPC1 to regulate Ca^2+^ entry, since specific deletion of CSD augments Ca^2+^‐store release‐induced Ca^2+^ influx in vascular endothelial and HEK‐293 cells (Sundivakkam et al. 2009). In addition, increased Cav1 expression in smooth muscle cells is associated with increased SOCE (Patel et al. 2007).

An increasing body of evidence indicates that SOCE is critical for maintaining sustained increases in [Ca^2+^]_cyt_ and in refilling Ca^2+^ into the ER in nonexcitable cells such as IECs (Brazer et al. 2003). The most significant finding reported in this study, however, is that Cav1 plays an important role in the stimulation of IEC migration after wounding by regulating [Ca^2+^]_cyt_ through SOCE. As shown in Figure 6, ectopic overexpression of Cav1 induced Cav1/TRPC1 complexes, enhanced Ca^2+^ influx, and stimulated cell migration over the denuded area after wounding. Consistently, HSG or MDCK cells overexpressing WT Cav1 also display a significant increase in SOC‐mediated Ca^2+^ influx (Lockwich et al. 2000; Kwiatek et al. 2006; Adebiyi et al. 2011). Our recent studies have demonstrated that induced STIM1 translocation to the PM promotes IEC migration after wounding by enhancing TRPC1‐mediated Ca^2+^ signaling (Rao et al. 2010, 2012). Although translocation of STIM1 from the ER/SR to the PM is a prerequisite for SOCE activation, STIM1 could not necessarily be translocated into the PM. In contrast, other proteins such as Cav1 may participate in the activation of SOCE channels (Brazer et al. 2003; Kwiatek et al. 2006). Our previous studies (Wang and Johnson 1991; Rao et al. 2001, 2007, 2012; Rao and Wang 2006) and others (Martin and Wallace 2006; Motawi et al. 2007; Seiler and Raul 2007) have also showed that cellular polyamines regulate gut epithelial restitution by modulating [Ca^2+^]_cyt_ homeostasis. Levels of cellular polyamines are rapidly increased after wounding and depletion of cellular polyamines decreases [Ca^2+^]_cyt_ due to the reduction of SOCE (Wang et al. 2000; Rao et al. 2001, 2002). It is not clear at present whether cellular polyamines are implicated in the regulation of Cav1 expression in the mucosal tissues after injury.

In summary, this study provides evidence indicating that target deletion of the *Cav1* gene delays gastric mucosal repair by reducing TRPC1‐mediated Ca^2+^ signaling. Our results obtained from an in vitro study further show that Cav1 physically interacts with TRPC1 and forms [Cav1/TRPC1] complexes, which is essential for the activation of TRPC1‐mediated Ca^2+^ influx after injury. Specific inhibition of Cav1 expression by transfection with the Cav1 siRNA prevents Cav1/TRPC1 association and thus decreasing SOCE in stable IEC‐TRPC1 cells. In contrast, overexpression of the *Cav1* gene increases Cav1/TRPC1 complex, induces SOCE, and stimulates cell migration after injury. Overall, these findings suggest that Cav1 functions as a novel regulator initiating TRPC1 activation after acute mucosal injury and plays an important role in the maintenance of GI mucosal integrity under biological and pathological conditions.

## Acknowledgments

J‐YW is a Senior Research Career Scientist, Medical Research Service, US Department of Veterans Affairs.

## Conflict of Interest

None declared.
